# Restless Legs Syndrome During Pregnancy and 12 Weeks Postpartum and Its Links to Cardiovascular Diseases, Stressful Life Events, and Psychiatric History

**DOI:** 10.3390/jcm9093046

**Published:** 2020-09-21

**Authors:** Tamme W. Goecke, Patricia Schnakenberg, Markus Frensch, Natalia Chechko

**Affiliations:** 1 Department of Gynecology and Obstetrics, Medical Faculty, Uniklinik RWTH Aachen University, 52074 Aachen, Germany; Tamme.Goecke@ro-med.de; 2 Department of Obstetrics, RoMed Hospital Rosenheim, 83022 Rosenheim, Germany; 3 Department of Psychiatry, Psychotherapy, and Psychosomatics, RWTH Aachen University, 52074 Aachen, Germany; pschnakenber@ukaachen.de; 4 Klinikum Mutterhaus der Borromäerinnen gGmbH, Abteilung für Gynäkologie u. Geburtshilfe, Feldstraße 16, 54290 Trier, Germany; markus.frensch@rwth-aachen.de; 5 Jülich Aachen Research Alliance (JARA)–Translational Brain Medicine, 52066 Aachen, Germany

**Keywords:** restless legs syndrome, pregnancy, stressful life events, cardiovascular diseases, psychiatric history

## Abstract

Restless legs syndrome (RLS) is highly prevalent among pregnant women. In the present study, a neurological–obstetrical sample of 561 postpartum women was retrospectively screened for RLS symptoms during pregnancy and in the first 12 weeks postpartum. The first screening took place within 1 to 6 days of delivery (T0) and the second 12 weeks after childbirth (T1). The pregnancy-related RLS prevalence rate was found to be 21% (n = 119), with the women suffering from RLS being more often affected by psychiatric history and having been more exposed to stressful life events. They were also found to have experienced baby blues more frequently shortly after childbirth. However, RLS in pregnancy did not appear to have any effect on the development of postpartum depression. Additionally, a positive trend was observed toward an association between pregnancy-related RLS and gestational diabetes and hypertension. Of the 119 women, 23 (19.3%) remained affected by RLS 12 weeks postpartum. Body mass index (BMI), weight gain, parity, childbearing history, or chronic stress exposure in pregnancy as measured by hair cortisol were not found to be linked to RLS. In summary, a comprehensive understanding of the interaction of clinical, environmental, and anamnestic factors can help shed valuable light on this pregnancy-related condition.

## 1. Introduction

Restless legs syndrome (RLS) is a common sensorimotor disorder with a 5–10% prevalence in the adult population [1], affecting roughly twice as many women as men [1,2]. The prevalence of RLS has been reported to be higher among the pluriparae [2,3,4,5,6], indicating that parity is a risk factor for RLS later in life [7]. For instance, a population-based study has found RLS prevalence in parous women to be much higher than in their nulliparous counterparts [2]. 

Frequently, RLS either appears for the first time during pregnancy or worsens during this period [8,9,10]; its main features occur during pregnancy, such as the quality of symptoms (e.g., an urge, triggered by unpleasant sensations, to move one’s legs), the circadian course (worse in the evening) and the motor activity that relieves the symptoms, resembling those of its idiopathic form [11]. 

Pregnancy-related RLS has been reported to be most prevalent in the third trimester (23%), gradually increasing from about 10% in the first trimester [8,9,10]. Although just prior to and after delivery the symptoms appear to alleviate, women with an incidence of RLS in pregnancy are highly likely to develop the chronic idiopathic form of this condition [7]. On the other hand, experience of RLS in a previous pregnancy and family history of RLS are strong predictors of the condition during an ongoing pregnancy [12]. Collectively, these observations suggest that genetic vulnerability play a role in the manifestation of pregnancy-related RLS [9]. The dramatic increases in estrogen and progesterone levels in pregnancy [13], insulin resistance [14], hypervolemia [13], weight gain [15] or increased iron use [16] can render genetically predisposed pregnant women additionally susceptible to the development of RLS symptoms. However, the available data in this respect are inconclusive [11,17,18,19,20,21,22]. Another intriguing aspect of RLS (both pregnancy-related and otherwise) is that it is frequently associated with conditions that are more debilitating than RLS itself. For instance, women with diagnosed idiopathic RLS have a higher risk of cardiovascular disease (CVD) mortality later in life relative to those without RLS [23]. In some studies, RLS in pregnancy has also been more frequently observed in women with pregnancy-related complications such as hypertension, preeclampsia or gestational diabetes [24,25].

While women with pregnancy-related RLS have been found to suffer from poorer health compared to their non-RLS counterparts, the evidence for specific disease associations is mixed. The findings of previous studies, however, raise the question whether pregnancy-related RLS symptoms may be early indicators of metabolic and cardiovascular problems later in life.

The aim of the present study was to estimate the prevalence, natural course, and specific risk factors of RLS in pregnancy and during the early postpartum period in this relatively large sample. As pregnancy is frequently associated with new manifestations or exacerbation of a CVD, the secondary purpose of the study was to investigate the link between gestational diabetes and gestational hypertension and pregnancy-related RLS. Conditions such as anemia, weight gain or hypothyroidism, which are frequently manifested during pregnancy, were also systematically looked at, as was the relationship between pregnancy-related RLS and postpartum mood, psychiatric history and history of stressful life events. Finally, the participants who were found to remain affected by RLS 12 weeks postpartum were identified in order to highlight the specific characteristics of the group and the risk factors that can potentially contribute to the condition persisting beyond childbirth. 

## 2. Methods

This study is part of an ongoing prospective longitudinal project aiming at early recognition of postpartum depression (PPD). The women were recruited in the Department of Gynecology and Obstetrics at the University Hospital Aachen within 1 to 6 days of childbirth (T0) between January 2016 and March 2020. The inclusion criteria were being aged between 18 and 45, being in the early postpartum period, and being healthy at the time of recruitment. The exclusion criteria of the study included alcoholic or psychotropic substance dependency or use during pregnancy, medication during pregnancy, neurological conditions, history of psychosis or manic episodes, depressive episodes in the current pregnancy, and inadequate proficiency in German or English. To control for the exclusion criteria, a brief non-standardized clinical interview was conducted according to the Diagnostic and Statistical Manual of Mental Disorders V (DSM V) criteria by an experienced psychiatrist (NC). A detailed sociodemographic description of the study population is provided in Table A1.

Following receipt of the participants’ informed consent, all participants were screened for RLS symptoms in pregnancy by means of the International Restless Legs Syndrome Rating Scale (IRLSS) [26] based on the criteria of the International Restless Legs Syndrome Study Group (IRLSSG) [26]. The IRLSS consists of 10 items, with scores ranging from 0 to 40. A score of 0–10 on this scale indicates mild RLS symptoms, a score of 11–20 indicates moderate, 21–30 indicates severe, and a score of 31–40 indicates very severe symptoms of RLS. 

At T0, in addition to the IRLSS, the Stressful Life Events Screening Questionnaire (SLESQ) [27] was used as a self-report instrument to help assess the participants’ encounter with 13 particular traumatic experiences, and the Edinburgh Postnatal Depression Scale (EPDS) [28] was used as a self-report instrument for the screening of PPD. The EPDS comprises 10 questions assessing depressive symptomatology in the postpartum period, with scores ranging from 0 to 30. Both IRLSS and EPDS are validated and widely used [27,29]. The participants were also required to complete a standardized questionnaire to help obtain anamnestic and pregnancy-related information, e.g., family history of psychiatric conditions, previous psychiatric experience, income, marital status, complications during pregnancy or birth mode. Additionally, a blood sample (one serum monovette of 7.5 ml) was collected to assess the thyroid-stimulating hormone (TSH), as well as the level of hemoglobin. A description of the sample characteristics can be found in Table A2.

**Figure 1:**
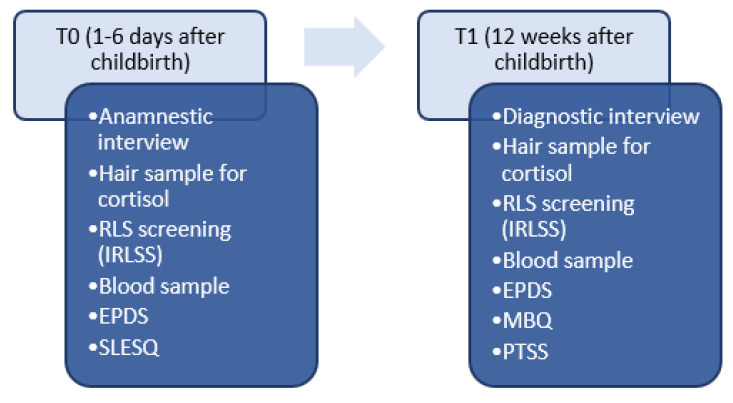
Study overview. RLS = Restless Legs Syndrome, IRLSS = International Restless Legs Syndrome Rating Scale, EPDS = Edinburgh Postnatal Depression Scale, SLESQ = Stressful life events screening questionnaire.

The second screening for postpartum RLS symptoms was carried out 12 weeks after delivery (T1). At T1 (12 weeks postpartum), the IRLSS and EPDS were applied for the second time. Further, in a subset of participants, the data on baby blues severity were assessed using the Maternity Blues Questionnaire (MBQ) [30]. These participants were also individually asked whether or not they had experienced any symptoms of baby blues. The presence and severity of premenstrual syndrome (PMS) symptoms were also assessed in a subset of participants by means of the Premenstrual Tension Syndrome Scale (PTSS) [31]. Again, a blood sample (one serum monovette of 7.5 mL) was collected to assess the thyroid hormone TSH (for an overview of the study procedure, see Figure 1).

The study was conducted in compliance with the Helsinki Declaration and was approved by the local ethics committee of the Medical Faculty, RWTH Aachen University, Germany.

### 2.1. Hair Sample Collection and Preparation 

Hair cortisol concentration (HCC) is widely regarded as a biomarker of chronic stress [32]. The participants’ hair samples were obtained from the posterior vertex of the head at T0 and T1, stored in aluminum foil, and treated as described in Stalder and Kirschbaum [33]. Here, 3 cm of hair was cut at each time point and approximately 50 mg was weighed in a polypropylene sampling tube using a microbalance. Hair samples were washed with 2-propanol, extracted with a four-fold deuterium isotope-labeled internal standard of cortisol and methanol for 24 h, and then analyzed with liquid chromatography triple-quadrupole mass spectrometry using an ion trap (Agilent Technologies 1200 Infinity Series QTRAP 5500 ABSciex). The limits of quantification were 0.05 ng/mL or 2 pg/mg hair, respectively.

### 2.2. Analysis

Independent sample T-tests (for parametric data) and Mann–Whitney-U tests (for non-parametric continuous variables), as well as chi-square tests (for categorical variables), were used to assess differences between the RLS group and control group. Within-group differences were analyzed by means of paired samples t-tests, and repeated-measures ANOVA was used to assess the course of RLS symptoms over time. Pearson correlations and Spearman correlations are reported for the parametric and non-parametric data, respectively. IBM Statistics 25 (SPSS, Chicago, IL, USA) was used for the analysis. For all statistics, a *p* 0.05 was considered significant.

## 3. Results

### 3.1. Course of RLS in Pregnancy in the Sample

In the present study, a neurological–obstetrical sample of 561 women in their early postpartum period were retrospectively screened for RLS symptoms during pregnancy. In the longitudinal study, the second screening was done 12 weeks postpartum. In total, 119 (21.2%) of the 561 women reported to have experienced RLS symptoms during pregnancy. While for 41 (34.5%) women this was the first pregnancy, for 73 (61.3%) women it was a=their second or third pregnancy. Of these 73 women, 34 (46.6%) had already experienced RLS symptoms during a previous pregnancy. Here, 85 women (77.3%) reported the RLS symptoms to have been most severe during the third trimester. The mean IRLSS score at T0 was M = 17.54, SD = 6.98 for all women experiencing RLS (n = 119).

### 3.2. Differences between the RLS and non-RLS Groups

The participants with RLS symptoms during pregnancy more often reported a psychiatric history (n = 32, 27.1%) compared to women without RLS (n = 79, 18.2%), χ^2^(1, 552) = 4.59, *p* = 0.032. In addition, a significantly larger number of women in the RLS group (n = 76, 63.9%) experienced stressful life events (SLE) compared to the control group (n = 208, 47.2%), χ^2^ (1, 561) = 10.46, *p* = 0.001. RLS in pregnancy, however, did not appear to have any effect on the development of a postpartum depression (PPD). 

**Figure 2:**
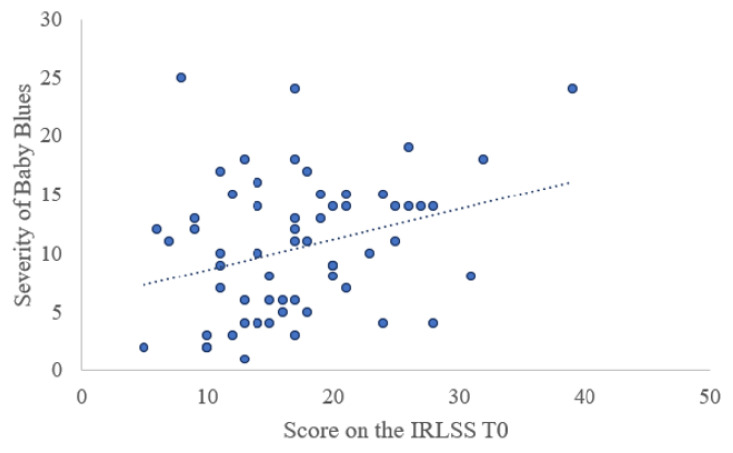
Correlation between IRLSS score at T0 and severity of baby blues (*n* = 61). IRLSS = International Restless Legs Syndrome Rating Scale, T0 = 1 to 6 days after childbirth.

**Figure 3:**
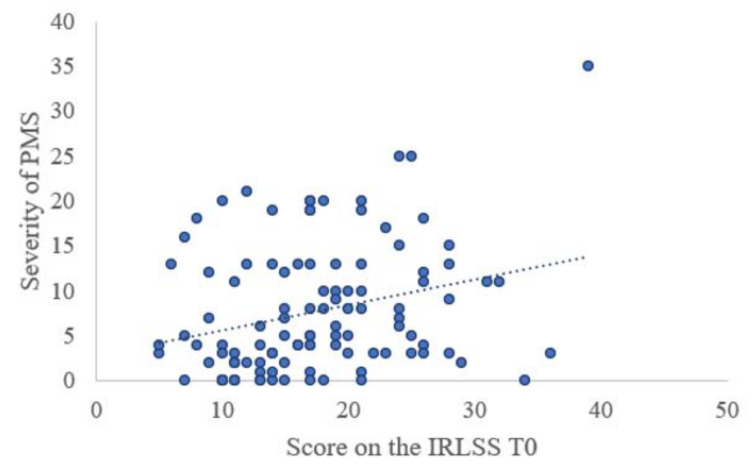
Correlation between IRLSS score at T0 and severity of PMS (*n* = 109). PMS = Premenstrual Syndrome, T0 = 1 to 6 days after childbirth.

More women with RLS experienced baby blues (n = 64, 53.8%) compared to women without RLS (n = 191, 43.5%), χ^2^ (1, 558) = 3.98, *p* = 0.046. The severity of baby blues was also found to be significantly linked to the severity of RLS symptoms (r = 0.296, *p* = 0.021) (Figure 2). Additionally, the severity of RLS (r = 0.260, *p* = 0.006) appeared to be affected by the severity of premenstrual syndrome (PMS) (Figure 3).

Group differences were observed also with regard to gestational diabetes, ᵪ^2^ (1, 561) = 3.71, *p* = 0.054. The differentiation between insulin-dependent gestational diabetes and dietary gestational diabetes showed women with RLS to be more often diagnosed with insulin-dependent diabetes (11.8%) compared to those without RLS (5.7%), ᵪ^2^ (3, 561) = 6.30, *p* = 0.098. Additionally, women in the RLS group were found to have experienced hypertension more often (7.6%) during pregnancy compared to their non-RLS counterparts (3.6%), ᵪ^2^ (3, 561) = 3.42, *p* = 0.064. A number of factors such as body mass index (BMI) (before and after pregnancy), weight gain during pregnancy, diagnosis of postpartum anemia and hemoglobin (Hb) value shortly after childbirth, diagnosis of hypothyroidism, TSH value, and hair cortisol concentration (HCC) at T0 and T1 were also considered, although none appeared to have any influence on RLS. The same applied to parity, birth mode, and birthweight of children (please refer to Table A3.).

### 3.3. Differences between Pregnancy-Related RLS and Persistent RLS

Of the 119 women, 23 (19.3%) continued to experience RLS 12 weeks after childbirth (persistent RLS), although the severity of the symptoms was lower at this stage (F (1,22) = 8.17, *p* = 0.009; T1, M = 16.04, SE = 1.47 vs. T0, M = 18.61, SE = 1.47). All of the others (n = 96 women, 80.7%) reported not to have experienced any RLS symptom following childbirth (pregnancy-associated RLS). Women with persistent RLS reported their RLS symptoms to be more severe (M = 2.26, SD = 0.85; IRLSS, question 8) at T0 compared to those with pregnancy-related RLS (M = 1.88, SD = 0.73), t (117) = −1.19, *p* = 0.030 (see Table A4.). The group with persistent RLS also reported, significantly more often than those with pregnancy-related RLS, that the severity of the symptoms was equally high over all three trimesters (χ^2^(3, 110) = 9.74, *p* = 0.021). Apart from that, women with persistent RLS did not differ from their counterparts with pregnancy-related RLS with respect to any other variable (see Table A5.).

## 4. Discussion

In a neurological–obstetrical sample of 561 postpartum women, we looked at various factors associated with pregnancy and the postpartum period of 12 weeks in the context of RLS. In our sample, the prevalence rate of the condition was found to be 21%. While in about 20% of those cases the symptoms were still reported 12 weeks postpartum (although mostly in weaker form than during pregnancy), in 80% of the cases the women experienced a complete recovery shortly after delivery. The majority of our participants (about 77%) experienced the RLS symptoms as most severe and most frequent in the last trimester of pregnancy. Women with persistent RLS, on the other hand, reported the symptoms to be equally severe in all trimesters of the pregnancy. Among those who had experienced RLS in a previous pregnancy, the symptoms in the current pregnancy showed a high tendency toward being repetitions of the ones experienced before (46.6%). These observations are in line with the results of previous studies on the prevalence and time course of pregnancy-related RLS [8,9]. 

In a comparison between the participants with and without RLS symptoms, those with pregnancy-related RLS were found to experience SLE more often than the ones without RLS. They were also more likely to have had a psychiatric history compared to their non-RLS counterparts (depression in 21.2% of the cases and anxiety in 3.4%). SLE is known to influence human behavior in multiple ways, often increasing the risk of suicide and depression [34,35,36]. In a study involving 169,373 participants, idiopathic RLS was found to be linked to an elevated risk of suicide and self-harm [37]. However, the mechanisms underlying the link between RLS and SLE, suicide, or self-harm remain unclear. Previous research has identified a potential link between child maltreatment and the development of somatic and visceral central sensitivity syndromes such as chronic pain, irritable bowel syndrome, and RLS [38]. Central sensitization has been thought to be related to an enhancement of the nociceptive neuronal pathways due to exposure to inflammation, injury, maltreatment, or other types of trauma [38]. Our findings indicate that SLE may contribute to central sensitization, causing RLS-like sensations, with multiple factors, including stress reactivity, mediating this association. In previous studies, symptoms of depression have been seen more frequently among participants with RLS compared to those without RLS [39,40,41,42,43]. Again, this link appears to be more prominent among women compared to men [40,44]. In a female-only Swedish study [44], for instance, women with RLS were found to be almost twice as likely to report depressed mood. Other studies have indicated that during times of hormonal change, women are at an increased risk of sleep disorders and RLS [45]. An increased prevalence of RLS has also been seen among women with vasomotor symptoms (night sweats) during the menopausal transition [44]. In our study, we found women with pregnancy-related RLS to experience baby blues more often than women without RLS. The RLS symptom severity also appeared to be influenced by the history of premenstrual syndrome (PMS). It must be noted here that both baby blues and PMS are associated with changes in the estrogen and progesterone levels. Women with RLS have shown higher levels of estradiol during pregnancy compared to control groups [46]. Although our observations, along with previous findings, underscore the role of hormonal fluctuation in the development of RLS in women, a causal link between changes in the hormone levels and RLS is yet to be determined. While pregnancy-related RLS may cause physical and psychological distress [47], chronic, mild stress can induce depression-like behavior related to dopaminergic serotonergic dysfunction [48]. As HCC helps determine long-term cumulative cortisol levels in order to characterize chronic stress exposure [32], we investigated the relationship between HCC levels during the last trimester and those 12 weeks postpartum, but did not find any association with respect to RLS diagnosis or RLS severity. Being the first study to investigate this relationship, the results suggest that long-term cumulative cortisol levels, as measured by HCC, are not linked to RSL in pregnancy. 

A positive association has previously been found between idiopathic RLS and CVD [49,50]. Our results, in line with previous findings, also indicate links between pregnancy-related RLS, gestational diabetes mellitus [51], and pregnancy-induced hypertension [22], with women suffering from insulin-dependent diabetes having been found to be more prone to pregnancy-related RLS. Collectively, these observations indicate a close link between CVD and RLS in general, and with pregnancy-related RLS in particular. A history of gestational diabetes seems to be associated with RLS later in life [25], underscoring a potential cardiovascular risk in the population affected by RLS.

While some studies have reported an association between lower Hb and pregnancy-related RLS [52,53], the link has not proved to be consistent [20,21]. In our study, a diagnosis of postpartum anemia or lower Hb was not found to be associated with RLS symptoms during pregnancy. However, the results of a number of neuroimaging studies indicate a reduction of brain iron content in RLS [54]. Of late, there has been a shift among researchers from the notion of dopamine deficiency in RLS to one indicating dopamine dysregulation possibly mediated through deficiencies in central nervous system (CNS) iron [55]. While this relationship likely underlies pregnancy-related RLS, the clinical diagnosis of anemia based on Hb alone may not be enough to determine any reduction of brain iron content.

As for the limitations of the study, there are three notable ones. First, the diagnosis of RLS was based solely on a self-report questionnaire (IRLSS), although it is a widely used measure in RLS diagnosis that possesses a good level of content validity [56]. Second, as the symptoms of RLS were assessed retrospectively, the risk of recall bias cannot be ruled out. Third, although the associations between baby blues and RLS, as well as the ones between PMS and RLS, are interesting and provide promising results, the data on baby blues and PMS were not assessed in the whole sample.

In conclusion, it can be stated that RLS in pregnancy is a heterogeneous syndrome with differences in onset timing. Our results indicate that this condition may also be linked to cardiovascular and metabolic diseases. Some of the clinical syndromes a woman experiences during pregnancy reappear later in life with the debilitating effects of ageing [55]. Thus, in some cases, RLS in pregnancy can be an early indicator of CVD or idiopathic RLS later in life. In our study, women suffering from RLS were found to be more affected by a psychiatric history and to have had a greater exposure to stressful life events. This suggests that alterations in stress response through multiple factors, such as history of stressful life events or psychiatric disorder, contribute to the development and manifestation of the condition. Interconnected in intricate ways, these factors are likely to contribute to the dysfunction of the mesolimbic and nigrostriatal dopaminergic pathways, triggering abnormalities in the limbic–nociceptive and sensorimotor networks [56]. Thus, RLS in pregnancy is a complex sensorimotor and neurological disturbance with major obstetrical and environmental contributions. More neuroimaging studies are needed for a better understanding of RLS in pregnancy and how it differs from its idiopathic form.

## Appendix A

**Table A1:** Sociodemographic data of the study population.

Variable	n (%)	M (SD)	Measured Study Range
Age	561 (100)	32.13 (4.79)	18–45
Married	413 (73.9)		
Unmarried	146 (26.1)		
Single Parent	16 (2.9)		
Number of Children			1–5
1	296 (53.0)		
2	187 (33.5)		
3	59 (10.6)		
4	13 (2.3)		
5	3 (0.5)		
Highest Education			
No school graduation	4 (0.7)		
Secondary school degree	23 (4.2)		
Junior high school degree	79 (14.5)		
University entrance	202 (37.1)		
College degree	195 (35.8)		
Doctoral degree	40 (7.4)		

**Table A2:** Description of the study population.

Pregnancy-Associated Characteristics	n (%)	M (SD)	Measured Study Range	Scale/Method Range
**Birth Mode**				
Spontaneous	307 (55.9)			
Ventouse	36 (6.6)			
Planned C-section	134 (24.4)			
Emergency C-section	72 (13.1)			
Twin birth	18 (3.2)			
**Pregnancy-Related Complications**				
Pre-eclampsia	11 (2.0)			
Eclampsia	2 (0.4)			
HELLP (hemolysis, elevated liver enzymes, and a low platelet count)	1 (0.2)			
Gestational Diabetes Mellitus	78 (13.9)			
Hypothyroidism	129 (23.0)			
Hypertension	25 (4.5)			
Premature birth	49 (8.7)			
Postpartum anemia	179 (31.9)			
Birth weight of child		3284.14 (567.94)	840–5350	
Days of gestation		272.88 (13.43)	192-294	
Breastfeeding T0	499 (89.0)			
Breastfeeding T1	432 (78.0)			
**RLS Characteristics**				
Restless Legs Syndrome (RLS) T0	119 (21.3)			
Severity of RLS symptoms in pregnancy (IRLSS score T0)		17.54 (6.98)	5–39	0–40
Restless Legs Syndrome (RLS) T1	23 (4.1)			
Severity of RLS symptoms 12 weeks postpartum (IRLSS score T1)		16.04 (7.77)	0–32	0–40
RLS in previous pregnancy	34 (6.1)			
**Psychiatric Characteristics**				
Depression Score T0 (EPDS)		5.49 (4.19)	0–22	0–30
Depression Score T1 (EPDS)		3.93 (4.16)	0–22	0–30
Stressful Life Events (SLESQ)	284 (50.7)			
Psychiatric history	111 (20.1)			
Depression	83 (15.0)			
Eating disorder	6 (1.1)			
Anxiety disorder	14 (2.5)			
Other	8 (1.4)			
**Laboratory Tests**				
HCC T0 (pg/mg)		9.84 (12.10)	0.58–91.55	2.2–72.2 ^1^
HCC T1 (pg/mg)		11.21 (79.58)	0.33–1468.22	2.2–72.2 ^1^
Hemoglobin (g/dL) T0		10.78 (1.48)	7.7–14.6	6.2–15.1
TSH T0		2.98 (1.56)	32–15.96	
TSH T1		1.44 (2.50)	01–33.87	

Notes: n = absolute frequency; % = relative frequency; M = mean; SD = standard deviation; Md = median; IRLSS= International Restless Legs Syndrome Scale; SLESQ = Stressful Life Events Screening Questionnaire; EPDS = Edinburgh Postnatal Depression Scale; HCC = hair cortisol concentration; T0 = baseline measure in childbed; T1 = measure 12 weeks after birth. ^1^ Reported range of the methodological validation study by [51].

## Appendix B

**Table A3:** Differences between women with RLS (n = 119) and women without RLS (n = 442).

Sociodemographic Characteristics	RLS (n = 119)	No RLS (n = 442)	*p*
	M (SD)	M (SD)	
**Age**	32.97 (4.75)	31.91 (4.79)	0.05
**Family Status**	**n (%)**	**n (%)**	0.05
Married	84 (70.6)	329 (74.8)	
Unmarried	35 (29.4)	111 (25.2)	
**Number of Children**			0.05
1	58 (48.7)	238 (54.2)	
2	41 (34.5)	146 (33.3)	
3	13 (10.9)	46 (10.5)	
4	6 (5.0)	7 (1.6)	
5	1 (0.8)	2 (0.5)	
**Highest Degree of Education**			0.05
No school graduation	1 (0.8)	3 (0.7)	
Secondary school degree	8 (6.8)	16 (3.8)	
Junior high school degree	14 (11.9)	65 (15.3)	
University entrance	39 (33.1)	163 (38.3)	
College degree	45 (38.1)	150 (35.2)	
Doctoral degree	11 (9.3)	29 (6.8)	
**Pregnancy-Associated Characteristics**			
**Birth Mode**			
Spontaneous	68 (59.1)	239 (55.1)	
Ventouse	6 (5.2)	30 (6.9)	
C-section	25 (21.7)	109 (25.1)	
Emergency C-section	16 (13.9)	56 (12.9)	
**Child Relocation after Birth**	36 (30.3)	119 (26.9)	0.05
**Pregnancy-Related Complications**			
Pre-eclampsia	1 (0.8)	10 (2.3)	0.05
Eclampsia	0 (0.0)	2 (0.5)	0.05
HELLP (hemolysis, elevated liver enzymes, and a low platelet count)	0 (0.0)	1 (0.2)	0.05
Gestational Diabetes Mellitus (GDM)	23 (19.3)	55 (12.4)	0.054
Insulin-dependent GDM	14 (11.8)	25 (5.7)	
Dietary-set GDM	9 (7.6)	28 (6.3)	
GDM not specified	0 (0.0)	2 (0.5)	
Hypothyroidism	34 (28.6)	95 (21.5)	0.05
Hypertension	9 (7.6)	16 (3.6)	0.064
Postpartum anemia	34 (28.6)	145 (32.8)	0.05
	**M (SD)**	**M (SD)**	
Length of pregnancy in days	272.46 (11.81)	272.99 (13.74)	0.05
Child’s birth weight (in gram)	3246.87 (534.43)	3294.17 (576.46)	0.05
	**n (%)**	**n (%)**	
Breastfeeding at T0	109 (91.6)	390 (88.2)	
Breastfeeding at T1	97 (82.9)	335 (76.7)	
RLS Characteristics			
Iron supplements	72 (60.5)	-	
RLS in previous pregnancy	43 (6.1)	-	
**Most severe RLS symptoms (*n* = 110)**			
First trimester	2 (1.8)	-	
Second trimester	8 (7.3)	-	
Third trimester	85 (77.3)	-	
Equally high over all trimesters	15 (13.6)	-	
**Severity of RLS Symptoms T0 (IRLSS)**			
Mild	19 (16.0)		
Moderate	63 (52.9)		
Severe	31 (26.1)		
Very severe	6 (5.0)		
**Psychiatric Characteristics**			
Depression score EPDS T0	6.27 (4.61)	5.29 (4.06)	0.023
Depression score EPDS T1	3.85 (3.81)	3.95 (4.25)	0.05
Stressful life events (SLESQ)	76 (63.9)	208 (47.2)	0.001
Baby blues	64 (53.8)	191 (43.5)	0.046
Severity of baby blues	10.52 (5.84)	9.29 (5.25)	0.05
Psychiatric history	32 (27.1)	79 (18.2)	0.032
Depression	25 (21.2)	58 (13.4)	
Eating disorder	2 (1.7)	4 (0.9)	
Anxiety disorder	4 (3.4)	11 (2.5)	
Other	1 (0.8)	6 (0.8)	
**Laboratory Tests**	***n* (%)**	***n* (%)**	
HCC T0 (pg/mg)	10.89 (14.14)	9.56 (11.52)	0.05
HCC T1 (pg/mg)	6.03 (5.81)	12.55 (11.52)	0.05
Hemoglobin (g/dL) T0	12.06 (1.12)	11.93 (1.18)	0.05
TSH T0	2.88 (2.04)	3.01 (1.37)	0.05
TSH T1	1.16 (0.69)	1.54 (2.87)	0.05

Notes: n = absolute frequency; % = relative frequency; M = mean; SD = standard deviation; Md = median; IRLSS = International Restless Legs Syndrome Scale; SLESQ= Stressful Life Events Screening Questionnaire; EPDS = Edinburgh Postnatal Depression Scale; HCC = hair cortisol concentration; T0 = baseline measure in childbed; T1 = measure 12 weeks after birth.

## Appendix C

**Table A4:** Differences between women with pregnancy-associated RLS and persistent RLS.

Variable	Pregnancy-Associated RLS (n = 96)	Persistent RLS (n = 23)	*p*
	M (SD)	M (SD)	
**Sociodemographic Characteristics**			
**Age**	33.07 (4.83)	32.52 (4.46)	0.05
**Family Status**			0.05
Married	66 (68.8)	18 (78.3)	
Unmarried	30 (31.3)	5 (21.7)	
**Number of Children**			0.05
1	45 (46.9)	13 (56.5)	
2	34 (35.4)	7 (30.4)	
3	11 (11.5)	2 (8.7)	
4	5 (5.2)	1 (4.3)	
5	1 (1.0)	0 (0.0)	
**Highest Degree of Education**			0.05
No school graduation	1 (1.1)	0 (0.0)	
Secondary school degree	8 (8.4)	0 (0.0)	
Junior high school degree	13 (13.7)	1 (4.3)	
University entrance	27 (28.4)	12 (52.2)	
College degree	35 (36.8)	10 (43.5)	
Doctoral degree	11 (11.6)	0 (0.0)	
**Pregnancy-Associated Characteristics**			
**Birth mode**			0.05
Spontaneous	55	13	
Ventouse	4	2	
C-section	22	3	
Emergency C-section	11	5	
**Child Relocation after Birth**	32 (33.3)	4 (17.4)	0.05
**Pregnancy-related complications**			0.05
Pre-eclampsia	1 (1.0)	0 (0.0)	
Gestational Diabetes Mellitus (GDM)			
Insulin-dependent GDM	10 (10.4)	4 (17.4)	
Dietary-set GDM	9 (9.4)	0 (0.0)	
Hypothyroidism	26 (27.1)	8 (34.8)	
Hypertension	6 (6.3)	3 (13.0)	
Postpartum anemia	29 (30.2)	5 (21.7)	
	**M (SD)**	**M (SD)**	
Length of pregnancy in days	272.48 (11.95)	272.35 (11.48)	0.05
Child’s birth weight (in gram)	3282.32 (548.51)	3098.91 (452.82)	0.05
	**n (%)**	**n (%)**	
Breastfeeding at T0	89 (92.7)	20 (87.0)	0.05
Breastfeeding at T1	78 (83.0)	19 (82.6)	0.05
RLS Characteristics			
Iron supplements	59 (61.5)	13 (56.5)	0.05
IRLSS score T0	17.27 (6.96)	18.65 (7.09)	0.05
IRLSS score T1	-	16.04 (7.77)	-
RLS in previous pregnancy	27 (28.1)	7 (30.4)	0.05
**Most severe RLS symptoms (n = 110)**			0.021
First trimester	2 (2.2)	0 (0.0)	
Second trimester	7 (7.8)	1 (5.0)	
Third trimester	73 (81.1)	12 (60.0)	
Equally high over all trimesters	8 (8.9)	7 (35.0)	
**Severity of RLS Symptoms T0 (IRLSS)**			0.05
Mild	2 (8.7)	17 (17.7)	
Moderate	12 (52.2)	51 (53.1)	
Severe	8 (34.8)	23 (24.0)	
Very severe	1 (4.3)	5 (5.2)	
**Psychiatric Characteristics**	**M (SD)**	**M (SD)**	
Depression score EPDS T0	6.01 (4.48)	7.35 (5.06)	0.05
Depression score EPDS T1	3.78 (3.86)	4.13 (3.67)	0.05
	**n (%)**	**n (%)**	
Stressful life events (SLESQ)	60 (62.5)	16 (69.6)	0.05
Baby blues	55 (57.3)	9 (39.1)	0.05
Severity of baby blues	10.67 (5.76)	9.67 (6.56)	0.05
**Psychiatric History**			0.05
Depression	19 (20.0)	6 (26.1)	
Eating disorder	1 (1.1)	1 (4.3)	
Anxiety disorder and depression	3 (3.2)	1 (4.3)	
Other	1 (1.1)	0 (0.0)	
**Laboratory Tests**	**M (SD)**	**M (SD)**	
HCC T0 (pg/mg)	11.06 (15.48)	10.31 (8.42)	0.05
HCC T1 (pg/mg)	6.13 (5.88)	5.74 (5.77)	0.05
Hemoglobin (g/dL) T0	10.74 (1.49)	11.07 (1.73)	0.05
TSH T0	2.74 (1.12)	3.70 (4.65)	0.05
TSH T1	1.17 (0.69)	1.11 (0.77)	0.05

Notes: n = absolute frequency; % = relative frequency; M = mean; SD = standard deviation; Md = median; IRLSS = International Restless Legs Syndrome Scale; SLESQ = Stressful Life Events Screening Questionnaire; EPDS = Edinburgh Postnatal Depression Scale; HCC = hair cortisol concentration; T0 = baseline measure in childbed; T1 = measure 12 weeks after birth.

## Appendix D

**Table A5:** The IRLSS questionnaire.

Question	All Women with RLS (n = 119)	Pregnancy-Associated RLS (n = 96)	Persistent RLS (n = 23)	Statistical Comparison between Pregnancy-Associated and Persistent RLS
	M (SD)			
IRLSS score T0 (Severity of RLS symptoms in pregnancy)	17.54 (6.98)	17.27 (6.96)	18.61 (7.06)	t (117) = 0.825, *p* 0.05
IRLSS score T1 (Severity of RLS symptoms 12 weeks postpartum)	3.39 (7.45)	-	16.04 (7.77)	
RLS in previous pregnancy	n = 34 (28.6%)	n = 27 (28.1%)	n = 7 (30.4%)	
Q1. How would you rate the RLS-related unpleasant sensations in your legs or arms?	2.04 (0.89)	2.01 (0.89)	2.17 (0.94)	t (117) = 0.784, *p* 0.05
Q2. How would you rate your urge to move because of the unpleasant sensations?	2.50 (0.99)	2.43 (0.98)	2.83 (0.98)	t (117) = 1.75, *p* 0.05
Q3. To what extent were the unpleasant sensations in your legs or arms alleviated by exercise?	1.83 (0.93)	1.82 (0.92)	1.87 (1.01)	t (117) = 0.215, *p* 0.05
Q4. How badly was your sleep affected by the unpleasant sensations?	1.80 (1.29)	1.77 (1.27)	1.91 (1.44)	t (117) = −2.13, *p* 0.05
Q5. How tired or sleepy were you during the day because of the unpleasant sensations?	1.32 (1.24)	1.34 (1.26)	1.22 (1.20)	t (117) = 0.470, *p* 0.05
Q6. Overall, how strong were the unpleasant sensations?	2.00 (0.86)	1.96 (0.86)	2.17 (0.89)	t (117) = −0.437, *p* 0.05
Q7. How often did the unpleasant sensations occur?	2.41 (0.96)	2.33 (0.98)	2.74 (0.81)	t (117) = 1.08, *p* 0.05
Q8. If you had unpleasant sensations, how severe were they on average?	1.95 (0.77)	1.88 (0.73)	2.26 (0.86)	t (117) = 2.20, *p* = 0.30
Q9. To what extent did your unpleasant sensations affect your ability to carry out daily activities?	0.65 (0.95)	0.70 (0.96)	0.43 (0.90)	t (117) = −1.19, *p* 0.05
Q10. How badly was your mood affected by the unpleasant sensations (making you, for example, angry, depressed, sad, anxious, or irritable)?	1.03 (1.13)	1.03 (1.11)	1.00 (1.24)	t (117) = −0.119, *p* 0.05
